# Gut Dysbiosis as a Shared Mechanism in Obesity and Hypertension: Exploring a Promising Therapeutic Avenue

**DOI:** 10.1002/edm2.70159

**Published:** 2026-03-28

**Authors:** Andrej Belančić, Almir Fajkić, Yusuf Ziya Sener, Ana Jelaković, Lejla Alić, Elvira Meni Maria Gkrinia, Donatella Verbanac, Bojan Jelaković

**Affiliations:** ^1^ Department of Basic and Clinical Pharmacology and Toxicology, Faculty of Medicine University of Rijeka Rijeka Croatia; ^2^ Department of Pathophysiology, Faculty of Medicine University of Sarajevo Sarajevo Bosnia and Herzegovina; ^3^ Department of Cardiology, Thoraxcenter Erasmus University Medical Center Rotterdam the Netherlands; ^4^ Department of Nephrology, Arterial Hypertension, Dialysis and Transplantation University Hospital Center Zagreb Zagreb Croatia; ^5^ University of Rijeka Faculty of Medicine Rijeka Croatia; ^6^ Department of Medical Biochemistry, Faculty of Medicine University of Sarajevo Sarajevo Bosnia and Herzegovina; ^7^ Independent Researcher Athens Greece; ^8^ Department of Medical Biochemistry and Haematology, Faculty of Pharmacy and Biochemistry University of Zagreb Zagreb Croatia; ^9^ School of Medicine University of Zagreb Zagreb Croatia

**Keywords:** gut microbiome, intestinal barrier dysfunction, metabolic syndrome, microbiome‐targeted therapy, obesity‐induced hypertension

## Abstract

**Background:**

Obesity and hypertension are interrelated global health challenges sharing common pathophysiological mechanisms, including insulin resistance, chronic inflammation and neurohormonal dysregulation. Emerging evidence highlights the gut microbiome as a crucial mediator in this interplay, influencing intestinal barrier integrity, systemic inflammation and metabolic homeostasis.

**Methods:**

In this narrative review, we critically examine the interplay between obesity‐induced hypertension and the gut microbiome, evaluating current evidence, therapeutic implications and future research priorities.

**Results:**

Obesity‐associated gut dysbiosis disrupts the intestinal epithelial barrier, increasing translocation of bacterial products like lipopolysaccharides into circulation, promoting systemic inflammation that exacerbates insulin resistance, adipose dysfunction and hypertension. Current treatments targeting obesity, from lifestyle modification to bariatric surgery, show beneficial effects on blood pressure, but microbiome‐targeted interventions are an evolving therapeutic frontier. Prebiotics, probiotics, synbiotics and faecal microbiota transplantation have demonstrated potential antihypertensive effects in preclinical and clinical studies, although findings are heterogeneous and require confirmation in larger randomised trials. Methodological challenges remain, including the need for advanced microbial sampling techniques beyond faecal analysis to fully capture disease‐relevant microbiota alterations.

**Conclusion:**

This review synthesises current knowledge on gut microbiome involvement in obesity‐induced hypertension, evaluates microbiome‐based therapeutic strategies and identifies critical research gaps to guide future investigations aimed at mitigating the dual pandemics of obesity and hypertension.

## Graphical abstract

This review synthesises current knowledge on gut microbiome involvement in obesity and hypertension, evaluates microbiome‐based therapeutic strategies, and identifies critical research gaps to guide future investigations aimed at mitigating the dual pandemics.

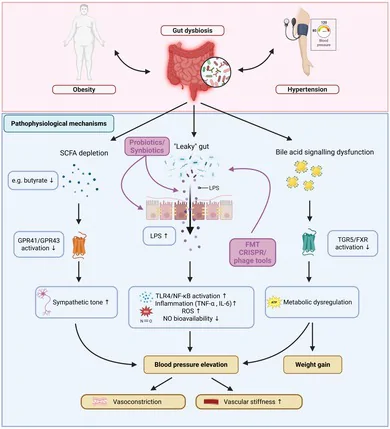

## 1 Introduction: Obesity, Hypertension and Their Overlapping Pandemics—A Call for New Perspectives

Obesity and hypertension represent two of the most formidable public health challenges of the 21st century—each driving a substantial share of global morbidity, mortality and healthcare costs, and increasingly recognised as interdependent epidemics [1]. An estimated 650 million people globally are living with obesity. Projections from the NCD Risk Factor Collaboration suggest that by 2030, nearly half of all adult men and women will have a high body mass index (BMI), with obesity prevalence reaching 17% in men and 22% in women [2]. These figures are not merely statistical abstractions; excess adiposity is implicated in a wide array of metabolic, mechanical and psychological complications, profoundly impairing quality of life and reducing life expectancy [3, 4].

Among the most prevalent and consequential complications of obesity is hypertension. Visceral adiposity is a critical determinant of blood pressure (BP) elevation, accounting for 65%–75% of essential hypertension cases, as evidenced by longitudinal data from the Framingham Heart Study [5]. Mechanistically, obesity‐induced hypertension is multifactorial, involving impaired pressure natriuresis from increased renal sodium reabsorption, direct renal compression by ectopic fat, activation of the renin–angiotensin–aldosterone system (RAAS), sympathetic nervous system (SNS) hyperactivity—possibly mediated by leptin and hypothalamic melanocortin signalling—and mineralocorticoid receptor activation independent of classic hormonal triggers [6].

Despite an expanding pharmacologic arsenal, hypertension control rates remain unacceptably low. Less than 45% of adults with hypertension currently achieve target BP levels, a decline that parallels rising obesity prevalence, population aging and the growing burden of treatment‐resistant hypertension [7, 8]. Both conditions are initially addressed with overlapping lifestyle interventions, including nutritional optimization, physical activity (PA) and stress reduction. However, one may also bear in mind that anti‐obesity agents such as liraglutide, semaglutide and tirzepatide have demonstrated ancillary antihypertensive effects, with systolic BP (SBP) reductions of 4.2–7.2 mmHg, while bariatric surgery may even result in remission [9, 10, 11, 12]. That said, even these advances fall short of fully addressing the complex pathophysiological interface between obesity and hypertension.

Emerging evidence highlights a novel and potentially modifiable player: the gut microbiome. Both obesity and hypertension are characterised by gut microbial dysbiosis, with overlapping alterations in bacterial composition, function and metabolite production [13]. These findings point to a ‘common soil’ hypothesis, wherein the microbiota may serve as a shared upstream determinant of cardiometabolic risk [14].

In this narrative review, we critically examine the interplay between obesity‐induced hypertension and the gut microbiome, evaluating current evidence, therapeutic implications and future research priorities. Our aim is to explore whether targeting the gut microbiome can represent a viable and innovative strategy in the management of this intertwined pandemic.

## 2 Microbial Dysbiosis in Obesity and Hypertension: A Common Soil Hypothesis

**FIGURE 1:**
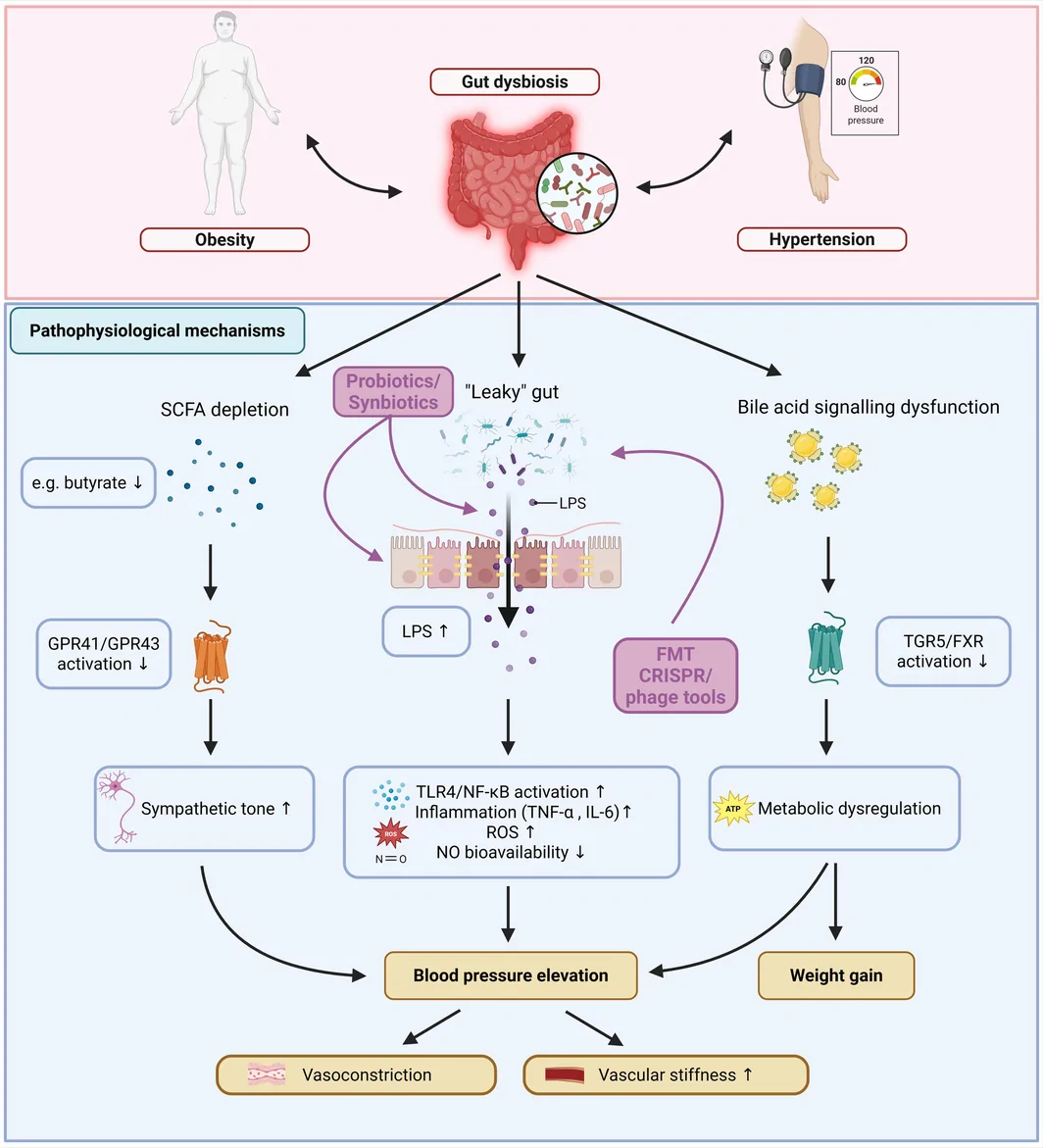
Overview of pathophysiological mechanisms and consequences of gut dysbiosis in relation to obesity‐induced hypertension. Purple squares represent potential sites of action of therapeutic options for gut dysbiosis. CRIPSR, clustered regularly interspaced short palindromic repeats; FMT, faecal microbiota transplant; FXR, farnesoid X receptor; GPR41/43, G‐protein coupled receptor 41/43; IL‐6, interleukin 6; LPS, lipopolysaccharide; NF‐κB, Nuclear factor kappa B; NO, nitric oxide; ROS, reactive oxygen species; SCFA, short chain fatty acids; TGR5, Takeda G protein‐coupled receptor 5; TLR4, Toll‐like receptor 4; TNF‐α, tumour necrosis factor α.

Hypertension and obesity have emerged as major global health pandemics, collectively responsible for millions of deaths worldwide. The pathogenesis of these conditions is multifactorial, involving an interplay of lifestyle factors, hemodynamic alterations, oxidative stress, hormonal dysregulation, insulin resistance and hyperinsulinemia, as well as genetic and epigenetic determinants [15]. In recent years, the gut microbiome has been increasingly recognised as a potential common denominator underlying both disorders. Accumulating evidence implicates gut microbiota dysregulation—commonly referred to as dysbiosis—as a causal contributor to the development and coexistence of hypertension and obesity. This effect appears to be mediated through complex interactions with the endocrine, gastrointestinal, nervous and immune systems, encompassing both immune‐dependent and immune‐independent mechanisms [16, 17]. Notably, the gut microbiome is now considered an integral pathophysiological factor in hypertension, in line with the multifactorial framework proposed by the mosaic theory of hypertension [18, 19]. Figure 1 presents an overview of pathophysiological mechanisms and consequences of gut dysbiosis in relation to obesity‐induced hypertension.

In obesity, experimental studies in mice report a reduction in bacterial diversity and an altered Firmicutes‐to‐Bacteroidetes (F/B) ratio, typically characterised by an increased abundance of Firmicutes and a decreased abundance of *Bacteroidetes phyla*. While this ratio is not universally consistent across all human studies due to various confounding factors like diet, geography and methodology, the overall consensus points to a less diverse and often functionally compromised gut microbiota, alongside changes in its overall composition [20, 21, 22]. Similarly, imbalances in the gut microbiome have been directly linked to the progression of hypertension. A study in stroke‐prone spontaneously hypertensive (SHR) rats has shown an increased F/B ratio as well as an increase in BP when their gut microbiota was transplanted into normotensive Wistar‐Kyoto rats [23, 24]. Furthermore, faecal microbiome transplantations (FMTs) from hypertensive human donors into germ‐free mice have demonstrated a direct increase in SBP and diastolic BP (DBP) in the recipient mice [23, 25]. Additionally, human studies involving nearly 7000 subjects have provided compelling evidence for the gut microbiota's causal role in hypertension, a finding that also reinforces the established links between an increased presence of Firmicutes and hypertension [26].

Crucially, gut microbiota‐derived metabolites, notably short‐chain fatty acids (SCFA), are increasingly acknowledged as key mediators through which the commensal microbiota modulates host homeostatic processes and systemic metabolic equilibrium, thereby potentially contributing to the pathogenesis of obesity‐associated hypertension. Indeed, SCFAs, such as acetate, butyrate and propionate, are fermentation products of dietary fibres by gut bacteria [27]. While SCFAs are generally considered beneficial for host health, their role in obesity is complex [27, 28]. An increased production of SCFAs in obese individuals might contribute to additional calorie harvest from the diet, potentially leading to weight gain [16, 29, 30, 31]. Importantly, the levels of SCFAs are dependent on the composition of the host's diet [32]. In the context of hypertension, SCFAs have mainly shown beneficial effects, especially acetate, primarily by activating G protein‐coupled receptors (GPCRs) and modulating immune cell activation [18, 33, 34, 35, 36, 37, 38]. Specifically, SCFAs exert their influence on the gut‐cardiorenal axis through endocrine effects, engaging with systems such as the RAAS [34, 35, 36]. Notably, SCFAs contribute to reducing the infiltration of pro‐inflammatory CD4^+^ and CD8^+^ T cells in the heart of hypertensive mice, while concurrently promoting an increase in regulatory T cells (Tregs), thus fostering an immunomodulatory environment conducive to BP regulation [33, 34, 38, 39, 40]. When interpreting the results of studies considering the role of SCFAs in obesity and hypertension, it is important to note that different types of fibres have different effects on the microbiota and consequently the types of SCFAs produced.

The gut microbiota plays a pivotal role in the biotransformation of primary bile acids (BAs), synthesised in the liver from cholesterol, into secondary BAs within the intestinal lumen. This microbial conversion, primarily through dihydroxylation, deconjugation and dehydrogenation reactions, yields a complex pool of secondary BAs, such as lithocholic acid and deoxycholic acid. Secondary BAs function as crucial signalling molecules that regulate host metabolism, immune function and cardiovascular homeostasis [41]. Their effects are largely mediated through the activation of specific nuclear receptors and GPCRs. Prominently, the farnesoid X receptor (FXR) and the Takeda G protein‐coupled receptor 5 (TGR5) are key receptors through which BAs exert their systemic effects [41, 42]. Interestingly, distinct gut bacteria in mice were reported to play a role in shaping their BA profiles and overall energy metabolism [43]. Consequently, altered BA metabolism due to gut dysbiosis significantly impacts glucose and lipid metabolism, energy homeostasis and fat deposition and contributes to increased adiposity [28, 42, 43, 44, 45]. These effects are mediated through signalling via FXR [44, 46, 47]. Similarly, the role of BAs in BP regulation has been shown in multiple studies. Activation of either TGR5 or FXR has been consistently shown to lower BP in various hypertensive animal models [48, 49]. Indeed, supplementation with primary and secondary BAs attenuated hypertension in mice models of hypertension [49, 50]. The mechanisms underlying these antihypertensive effects are multifaceted, encompassing vasodilation and modulation of immune cell function, specifically influencing Treg cell differentiation [49, 50, 51].

The intestinal epithelial barrier is crucial for maintaining host health, as it selectively allows for nutrient absorption while blocking harmful substances [52]. The gut microbiome maintains this barrier, and gut dysbiosis is a key driver of barrier dysfunction through multiple mechanisms, including impairing the protective mucus layer, reducing tight junction integrity (leading to ‘leaky gut’), decreasing vital SCFA‐producing bacteria, and increasing pathobionts that directly damage epithelial cells and induce inflammation [53, 54, 55]. In obesity, intestinal barrier dysfunction is common, often linked to high‐fat diets that alter gut microbiota [56]. This leads to increased bacterial lipopolysaccharide (LPS) translocation, where a compromised barrier allows more LPS to enter systemic circulation [57, 58]. Systemic LPS exposure then triggers chronic low‐grade inflammation by activating TLR4, which drives insulin resistance, adipose tissue dysfunction and metabolic syndrome—all related to obesity [59, 60]. This LPS‐induced inflammation and metabolic dysregulation ultimately contribute to energy imbalance, promoting fat accumulation [60]. Similarly, barrier dysfunction contributes to the development of hypertension. A ‘leaky gut’ in hypertensive individuals allows LPS and other bacterial products to translocate, causing chronic low‐grade systemic inflammation [61, 62, 63, 64]. This inflammation contributes to vascular dysfunction, endothelial damage and arterial stiffness. Indeed, increased plasma LPS levels and barrier dysfunction markers are observed in essential hypertension [34, 65, 66]. In summary, the resulting increased systemic exposure to bacterial products and chronic inflammation drives metabolic dysregulation in obesity and cardiovascular dysfunction in hypertension.

SNS activation, alongside neurohumoral changes and gut microbiome dysbiosis, is a key component of the complex interactions that lead to obesity‐induced hypertension. Excess adipose tissue heightens SNS activity, notably renal sympathetic outflow, promoting sodium reabsorption and RAAS activation [67]. Leptin further amplifies SNS stimulation [68]. Neurohumoral shifts, including elevated anti‐natriuretic hormones and deficient natriuretic peptides, contribute to sodium retention and elevated BP, with inflammation and immune activation modulating these mechanisms [67, 68]. Additionally, SNS hyperactivity impairs intestinal barrier integrity, increasing permeability and fostering inflammation, exacerbating dysbiosis. This dysbiosis then boosts circulating LPS and microbial metabolites, stimulating sympathetic responses and systemic inflammation, thereby creating a vicious cycle that sustains hypertension [61, 62, 69]. Thus, bidirectional communication via the microbiota‐gut‐brain axis is central, as SNS alters gut microbiota, and microbial metabolites modulate neurohumoral and immune responses impacting BP. Indeed, experimental models confirm the causal role of gut dysbiosis in neurogenic hypertension [62, 69, 70]. RAAS blockade, for instance with losartan, can restore gut microbiota composition, improve intestinal barrier integrity and lower BP, partially through microbiome‐mediated mechanisms. Furthermore, FMT from RAAS‐blocked animals to hypertensive recipients can transfer these beneficial effects [70]. Overall, a vicious cycle of inflammation and impaired intestinal barrier function perpetuates obesity‐induced hypertension via the microbiota‐gut‐brain axis.

In summary, current research strongly suggests that alterations in the gut microbiota contribute to the development of hypertension in obese individuals through mechanisms involving microbial metabolites, inflammation, immune dysregulation, impaired gut barrier function and irregularities of the microbiota‐gut‐brain axis. This understanding opens up new avenues for potential therapeutic interventions targeting the gut microbiome to manage obesity‐related hypertension.

## 3 Current Therapeutic Strategies in Obesity and Their Impact on Hypertension

Obesity and hypertension are intimately intertwined pathophysiological entities, frequently coexisting and sharing overlapping mechanisms involving insulin resistance, sympathetic overactivation, chronic inflammation and altered neurohormonal regulation. Contemporary therapeutic approaches for obesity, ranging from lifestyle interventions to pharmacologic therapy and bariatric surgery, not only yield substantial weight reduction but also exert clinically meaningful reductions in BP, effectively addressing two major cardiovascular risk factors simultaneously.

Lifestyle modification remains the cornerstone of obesity and hypertension management. Meta‐analytic evidence demonstrates that even modest weight loss is associated with significant BP reductions: a mean BMI decrease of 2.27 kg/m^2^ correlates with a clinic SBP reduction of 5.79 mmHg and DBP by 3.36 mmHg, while a larger BMI reduction of 4.12 kg/m^2^ leads to even greater BP declines (SBP −6.65 mmHg, DBP −3.63 mmHg) [71]. These effects are magnified in individuals achieving ≥ 3 kg/m^2^ reduction, underscoring a dose–response relationship between weight loss and BP control. Diet‐based strategies, particularly those emphasising increased fruit and vegetable intake, reduced sodium and adherence to Mediterranean dietary patterns, are similarly effective. Arnotti et al. confirmed that these dietary interventions lower SBP and DBP significantly in overweight and obese individuals, contributing to reduced cardiovascular risk [72]. Furthermore, PA exerts multifaceted benefits beyond weight reduction. As per the Physical Activity Guidelines Advisory Committee, PA not only reduces incident hypertension but also lowers BP across the spectrum of normotensive to hypertensive individuals, with the most pronounced effects observed in those with prehypertension. These findings illustrate how integrative lifestyle strategies, though often modest in isolation, can synergistically yield substantial BP benefits when sustained and combined [73].

Chronic stress activates the SNS and the hypothalamic–pituitary–adrenal axis, promoting inflammation, dysregulated cortisol secretion and metabolic disturbances such as hyperinsulinemia. These physiological alterations, compounded by adverse behavioural factors including poor sleep, depression and social isolation, exacerbate cardiovascular risk and complicate weight management. Effective stress reduction through regular exercise, relaxation techniques such as meditation and deep breathing, social support and professional counselling plays a pivotal role in improving psychological well‐being and thereby contributes to lowering BP and facilitating weight loss [74].

In cases of inadequate response and clinical indication for more intensive treatment, pharmacotherapy offers a compelling adjunct. Glucagon‐like peptide‐1 (GLP‐1) receptor agonists, including liraglutide and semaglutide, as well as the dual GLP‐1/GIP receptor agonist tirzepatide, have revolutionised obesity pharmacotherapy with concurrent BP‐lowering effects [9, 10, 11]. In randomised controlled trials (RCTs), semaglutide and tirzepatide significantly reduced SBP by 5.10 mmHg (−6.16 mmHg vs. −1.06 mmHg for placebo) and 6.2 mmHg (−7.2 mmHg vs. −1.0 mmHg for placebo), respectively, alongside substantial weight loss of 14.9% with semaglutide and 20.9% with tirzepatide [10, 11]. These pharmacologic agents modulate not only appetite and satiety centers but also favourably impact metabolic and cardiovascular parameters, potentially through mechanisms involving natriuresis, improved endothelial function and reduced arterial stiffness. Notably, these benefits occur with relatively few adverse hemodynamic effects, making them attractive options in patients with coexisting obesity and hypertension.

At the most intensive end of the therapeutic spectrum, bariatric surgery—particularly Roux‐en‐Y gastric bypass and sleeve gastrectomy—yields the most profound and durable effects on both weight and BP. The GATEWAY trial demonstrated that bariatric surgery leads to a 45.8%–51.0% remission rate of ambulatory or office‐based arterial hypertension [12]. Furthermore, a systematic review by Climent et al. confirmed that gastric bypass confers a superior rate of hypertension remission at both one and 5 years post‐operatively (RR 1.14–1.26) compared to sleeve gastrectomy, despite similar BP reductions between procedures [75]. These effects appear independent of weight loss alone, suggesting metabolic and neurohumoral modulation as additional contributors. Thus, from lifestyle changes to surgical interventions, therapeutic strategies for obesity are consistently associated with significant antihypertensive effects, highlighting a unifying treatment axis that concurrently addresses two interdependent pathologies.

Emerging research also suggests that these interventions—particularly dietary modification, GLP‐1‐based therapies and bariatric surgery—modulate the gut microbiome, a critical regulator of host metabolism, inflammation and vascular tone. Shifts in microbial composition, diversity and metabolite production following these treatments have been implicated in sustaining weight loss and improving BP regulation, setting the stage for microbiome‐targeted interventions as a promising future strategy. By addressing obesity, we may indeed be treating hypertension at its roots, killing two birds with a single, evidence‐based stone.

## 4 Gut Microbiome‐Targeted Therapeutics: Mechanistic Rationale and Emerging Approaches

Obesity‐induced hypertension is a condition that is increasingly attributed to, in addition to metabolic and neurohormonal factors, microbial imbalances in the gut. A low diversity of gut dysbiosis, characterised by the loss of beneficial bacteria, can contribute to low‐grade systemic inflammation, as well as endothelial dysfunction and sympathetic overactivity, which in turn may fuel BP elevation in obesity; this might be the underlying cause. Building on this mechanistic insight, several microbiome‐targeted therapeutic strategies have gained attention. The current microbiome editing tools used for hypertension primarily include probiotics, prebiotics, synbiotics, dietary interventions and FMT. These approaches aim to modulate gut microbial composition and function to influence BP regulation. Additionally, more advanced approaches such as engineered microbial consortia aim to reestablish a healthy microbial community with sustained metabolic benefits. Together, these interventions offer promising avenues for restoring microbial balance and interrupting the pathogenic loops that link gut dysbiosis to hypertension in the context of obesity.

### 4.1 Prebiotics

Prebiotics have been shown to influence gut dysbiosis within the context of obesity and its related comorbidities, such as hypertension. A body of evidence from animal and in vitro studies demonstrates that prebiotic fibres like inulin, fructooligosaccharides (FOS), galactooligosaccharides (GOS) and poly‐D‐3‐hydroxybutyrate (PHB) are capable of modulating the composition of gut microbiota, increasing the population of good bacteria, for example, Bifidobacteria, *Lactobacillus* as well as SFCA producers and improving gut barrier function in models for obesity and metabolic syndrome [76, 77]. Mechanistically, prebiotics are fermented by gut microbiota to generate SCFAs (acetate, propionate and butyrate), which exert antihypertensive effects by activating GPCRs such as GPR41, GPR43, GPR109A and Olfr78, located in vascular, renal and central nervous system tissues. GPR41 and Olfr78 exert opposing effects on BP regulation, while GPR43 and GPR109A are associated with immune modulation and cardiac protection. Deficiency of prebiotic fibre reduces SCFA levels, leading to insufficient GPCR activation and the emergence of a pro‐hypertensive microbiota [33, 78].

In animal models of metabolic syndrome and obesity‐induced hypertension, inulin supplementation ameliorated hypertension and cardiac injury. It is associated with the reduction of systemic inflammation and improvement in gut dysbiosis; however, it does not affect obesity or insulin resistance directly [76]. High‐fat diet‐induced obese mice received prebiotic supplementation with FOS and GOS that reduced dysbiosis, increased acetate‐producing bacteria, improved intestinal permeability and decreased both peripheral and central inflammation. It is mechanistically linked to the pathophysiology of obesity‐induced hypertension [79]. Other prebiotics like PHB have also been proven to increase beneficial gut bacteria while lowering dyslipidemia in obese mice, which further supports the role of prebiotics regarding modulation available for gut microbiota as well as metabolic risk factors [80].

Prebiotics also influence the RAAS and SNS activity. In perinatal high‐fat diet models, maternal prebiotic supplementation prevented hypertension in offspring by modifying microbial composition, lowering levels of pro‐hypertensive metabolites like trimethylamine‐N‐oxide (TMAO), and altering renal *RAS* gene expression [81]. Furthermore, prebiotics reduce neuroinflammation and oxidative stress in central autonomic centers such as the hypothalamic paraventricular nucleus, contributing to BP regulation [82].

In vitro studies on faecal cultures from obese subjects proved that different prebiotics are capable of modulating the microbiota composition by shifting it to a profile characterised by metabolic health; however, the magnitude and nature of these effects may be quite different and depend on the particular prebiotic as well as the baseline microbiota of the host. Such microbiota changes have been associated with improvements in metabolic and inflammatory parameters relevant to obesity‐induced hypertension [83, 84, 85]. Additionally, prebiotics help preserve gut barrier integrity and reduce gut‐derived inflammation, a key contributor to the development of hypertension. In models of obstructive sleep apnea‐induced hypertension, prebiotic supplementation restored SCFA‐producing bacteria, preserved the mucus barrier, increased cecal acetate and prevented both gut and brain inflammation [86].

Taken together, prebiotics exert multifactorial benefits in obesity‐induced hypertension by promoting SCFA production, activating metabolite‐sensing receptors, modulating inflammation and immune responses, influencing neurohumoral systems and maintaining gut barrier function.

### 4.2 Probiotics

Probiotics can be considered an emerging therapeutic strategy for modulating dysbiotic guts in obesity‐induced hypertension. Their effects are multifactorial, spanning from reshaping microbial composition to improving systemic metabolic parameters through gut barrier restoration, microbial metabolite production and immune regulation. A growing body of preclinical and clinical evidence supports their efficacy in restoring eubiosis and attenuating cardiovascular risk [63, 87, 88]. At the microbial level, probiotic supplementation consistently reduces the F/B ratio and increases the presence of health‐related groups such as *Bifidobacterium*, *Lactobacillus* and *Akkermansia muciniphila* [63, 87]. These compositional shifts result in greater changes related to the production of more SFCAs, especially butyrate, propionate and acetate, which have vasoprotective and anti‐inflammatory effects, thereby improving gut barrier activity [88].

Mechanistically, probiotics stimulate mucin secretion and the expression of tight junction proteins, such as MUC2, occludin, claudins and ZO‐1, thereby strengthening gut barrier integrity and preventing the translocation of bacterial components, including LPS [89]. By reducing intestinal permeability and systemic endotoxemia, probiotics attenuate LPS/TLR4‐mediated activation of pro‐inflammatory pathways (NADPH oxidase, MAPK and NF‐κB), which are critical contributors to vascular inflammation and endothelial dysfunction [64]. Additionally, probiotics promote immune homeostasis by restoring the balance between Th17 cells and Tregs, increasing Treg infiltration into vascular tissues, and downregulating pro‐inflammatory cytokines such as TNF‐α and IL‐6 [89]. The elevation in SCFAs further supports these effects by activating GPCRs (e.g., GPR41, GPR43) on vascular and immune cells, enhancing nitric oxide (NO) bioavailability and modulating SNS tone and renal sodium handling [88].

Furthermore, probiotics may reduce levels of pro‐hypertensive microbial metabolites such as trimethylamine (TMA) and its hepatic derivative TMAO, which are known to impair endothelial function and elevate BP [90].

Several strains have shown strain‐specific benefits in experimental and clinical settings. *Bifidobacterium breve CECT7263* and *Lactobacillus fermentum CECT5716* have shown antihypertensive effects in rodent models mediated by modulation of gut microbiota, augmentation of SCFA‐producing bacteria and improved endothelial function [91]. In human trials, *Lacticaseibacillus paracasei MSMC39‐1* and *Bifidobacterium animalis TA‐1* not only reduce SBP, BMI and waist circumference but also improve the abundance of genera like *Blautia*, *Roseburia* and *Ruminococcus*, which are metabolic‐health‐related microbes [92].

Next‐generation probiotics also open novel therapeutic approaches. *Limosilactobacillus reuteri* and *balticus* proved effective in limiting weight gain and systemic inflammation in diet‐induced obese mice by modulating the major microbial families and metabolic pathways [93]. *Levillactobacillus* brevis D17, a GABA‐producing strain, was very effective in improving glucose and lipid metabolism along with enrichment of *Muribaculaceae*, *Bifidobacterium globosum* and other taxa [94]. In another study, *Bifidobacterium adolescentis FJSSZ23M10* and *Lactobacillus plantarum K50* increased butyrate levels, corrected the F/B ratio and inhibited pro‐inflammatory markers in animal models [64, 95].

These mechanisms eventually translate into tangible health benefits at the clinical level. RCTs have proven that multi‐strain probiotic supplementation for hypertensive and obese patients improves BP control, insulin sensitivity, lipid metabolism and systemic inflammatory markers, yielding significantly better results when combined with dietary interventions. The lower prevalence of obesity and hypertension observed in observational studies is associated with regular probiotic consumption, further supporting these findings [96, 97].

### 4.3 Synbiotics

Current evidence from preclinical studies suggests that synbiotics—combinations of probiotics and prebiotics—can positively modulate dysbiotic gut flora under conditions of obesity‐induced hypertension and metabolic derangements. Multiple animal models of diet‐induced obesity and metabolic syndrome have shown that synbiotic interventions restore microbial diversity and composition by enriching beneficial genera such as *Bifidobacteria*, *Lactobacilli*, *Akkermansia*, *Faecalibacterium* and *Roseburia*, while reducing potentially pathogenic bacteria including *Enterobacteriaceae* and certain *Clostridium* species [98]. These microbiota changes are associated with improvements in metabolic parameters, including reductions in body weight, abdominal fat mass, insulin resistance and systemic inflammation, as well as enhanced gut barrier integrity and decreased circulating LPS levels [99, 100, 101].

In terms of hypertension, synbiotic supplementation in high‐fat diet‐induced models of metabolic syndrome has been shown to reduce elevated BP, primarily by restoring NO bioavailability, especially through normalisation of the neuronal NO synthase–protein kinase A signalling pathway in vascular tissues [102]. This leads to a reduction in vascular dysfunction associated with gut microbiota and its metabolite‐modulating factors [103]. Additionally, activation of peroxisome proliferator‐activated receptors (PPARβ and PPARγ) in the ileum has been demonstrated as a key signalling axis in synbiotic‐mediated antihypertensive effects, independent of the RAAS. Metabolomic data also suggest upregulation of beneficial lipids (e.g., lysophosphatidylethanolamine, phosphatidylcholine), further supporting vascular and metabolic homeostasis [104].

Synbiotics reduce systemic levels of pro‐inflammatory cytokines, particularly TNF‐α and IL‐6, and increase the production of SCFAs. In parallel, synbiotics inhibit the hepatic TLR4/NF‐κB signalling cascade, a pathway linked to metabolic endotoxemia and hypertension, and reduce inflammation in adipose tissue and liver [105]. Intestinal barrier improvement is also a prominent mechanism; synbiotics enhance tight junction protein expression, reduce gut permeability and suppress β‐catenin expression in intestinal epithelium, thereby limiting systemic exposure to inflammatory microbial products [106].

Synbiotic interventions have shown modest but statistically significant reductions in SBP, particularly in adults with hypertension or metabolic syndrome. The antihypertensive effect appears to be more remarkable in individuals less than 50 years old with a BMI of < 30 kg/m^2^, and it should last at least 12 weeks of supplementation, besides its effects on DBP, which are still inconsistent [107]. Synbiotics in patients with metabolic syndrome lower the SBP by about 1.8 mmHg. Improvements were also observed in terms of insulin levels, triglycerides and waist circumference [108]. However, data seem to indicate that individuals with non‐alcoholic fatty liver disease (NAFLD) will not benefit from it since no significant effect on either SBP or DBP has been observed so far [109]. Overall, while the absolute decrease in SBP is moderate (1–3 mmHg), the advantage may be significant within the context of broader cardiometabolic risk reduction, especially in younger, non‐obese individuals.

Collectively, these findings indicate that synbiotics target multiple mechanistic pathways relevant to obesity‐induced hypertension and these pleiotropic effects position synbiotics as a promising adjunctive strategy for the management of obesity‐associated hypertension.

### 4.4 Faecal Microbiota Transplantation

FMT has been shown in preclinical and clinical studies to modulate gut dysbiosis associated with obesity and its metabolic sequelae, including hypertension.

Animal studies indicate that transplantation of gut microbiota from obese or hypertensive donors transmits cardiovascular risk phenotypes, such as increased arterial stiffness, elevated SBP and metabolic abnormalities, to normotensive recipients [110]. Conversely, FMT from lean or metabolically healthy donors leads to improved gut microbial diversity, expansion of SCFA‐producing taxa (e.g., *Bacteroidetes*, *Prevotella*, *Clostridia*), and reduction in pro‐inflammatory genera (e.g., *Eggerthella lenta* , *Erysipelatoclostridium ramosum*), with decreased expression of pro‐inflammatory cytokines such as IL‐6 and TNF‐α [111, 112, 113]. FMT also modulates host metabolism via alteration of amino acid and BA profiles, and attenuates tissue‐specific inflammation by affecting macrophage activation states in adipose and vascular tissues [114].

In humans, systematic reviews and meta‐analyses of RCTs have shown that FMT can lead to significant changes in the composition of gut microbiota in patients with obesity and metabolic syndrome. It may also improve unhealthy elevated BP, insulin resistance and abnormal lipid profiles; however, the clinical significance and durability of these effects are unknown [115, 116]. Multiple recent meta‐analyses have confirmed the short‐term benefits of FMT on metabolic parameters, such as reductions in fasting glucose and insulin, and modest increases in high‐density lipoprotein cholesterol, typically observed within 2–6 weeks post‐intervention. However, these effects are generally small and not sustained in the long term, and FMT shows no significant impact on weight reduction or durable metabolic control [115, 116, 117, 118].

In a recent multicenter, RCT in hypertensive patients, it was found that FMT led to transient reductions in SBP along with significant shifts in gut microbial richness, the most salient being changes in the abundance of key taxa (e.g., *Firmicutes*, *Bacteroidetes*, *Parabacteroides*, *Prevotella*, *Bacteroides*) that correlated with BP and metabolite profiles. However, the antihypertensive effect was not sustained beyond the initial weeks following the intervention [119].

Additional studies in overweight or diabetic populations suggest that FMT may yield greater cardiometabolic benefits when combined with dietary modifications; however, such findings are limited by small sample sizes, non‐blinded designs and a lack of long‐term follow‐up. Accordingly, current systematic reviews emphasise that FMT remains an investigational strategy and cannot yet be recommended for routine clinical use in metabolic syndrome or hypertension management [120, 121].

In summary, FMT can be considered a mechanistically rational, yet currently investigational, method for modulating gut microbiota in hypertension induced by obesity. It leads to short‐term improvement in metabolism and vasculature with moderate, transient and context‐dependent effects on BP. The future of translating FMT into an effective cardiometabolic therapy lies in helping to increase the durability of microbial engraftment, along with the optimization of host–donor matching.

### 4.5 Dietary Interventions

Dietary interventions should be viewed as primary in the modulation of gut dysbiotic features within obesity‐induced hypertension, as available evidence supports their potential to alter microbial composition and downstream cardiometabolic outcomes. High‐fibre and hypocaloric diets have been shown to beneficially shift the gut microbiota among obese and metabolic syndrome individuals with hypertension. Such interventions would aim to increase more generous genera, such as *Lactobacillus* and *Bifidobacterium*, thereby lowering the F/B ratio—a dysbiotic signature reported in both obesity and hypertension. These microbial changes are accounted for by corresponding improvements in BP, anthropometric parameters and metabolic biomarkers, therefore implying a mechanistic linkage between dietary modulation of microbiome contribution to the amelioration of features related to hypertension [82].

Preclinical models also demonstrate that dietary patterns rich in fermentable fibre, polyphenols and omega‐3 polyunsaturated fatty acids (PUFAs) can reverse gut dysbiosis induced by a high‐fat diet. These interventions consistently reduce the F/B ratio and provide a more conducive environment for the proliferation of beneficial taxa such as *Akkermansia muciniphila* , *Bifidobacterium*, *Lactobacillus*, and hence increase SCFA production. SCFAs, particularly butyrate, are associated with improving gut barrier integrity, reducing systemic inflammation and maintaining normal BP regulation [122, 123].

Omega‐3 PUFAs, in particular, suppress pro‐inflammatory and endotoxemia‐associated bacteria (e.g., *Desulfovibrio*, *Lachnoclostridium*), further supporting their anti‐hypertensive potential via microbiota modulation [124].

Comprehensive reviews emphasise that diet‐based approaches, such as the Mediterranean and DASH (Dietary Approaches to Stop Hypertension) diets, as well as more general whole‐grain, fibre and plant‐based food intakes, consistently promote eubiotic microbiota profiles and are associated with better control of BP and metabolic parameters, apart from weight loss [125]. Conversely, Western diets, unhealthy patterns high in saturated fat and low in dietary fibre, worsen dysbiosis, create an inflammatory environment and contribute to the pathogenesis of hypertension [122, 123].

### 4.6 The Novel Microbiome Editing Tools

To date, the use of Clustered Regularly Interspaced Short Palindromic Repeats (CRISPR)–Cas9 technology in hypertension therapy is primarily experimental and mostly restricted to studies on preclinical models. For example, gene editing of host targets, such as GPER1, in hypertensive rats resulted not only in BP normalisation but also caused shifts in gut microbiota, which opens up the possibility of a microbiome‐mediated mechanism [126]. Although theoretically, CRISPR tools can be used for manipulating microbial compositions by removing particular strains or changing microbial gene expression, their use for directly altering the microbiome toward an effect on hypertension is at such an early stage that there has been no clinical translation [127]. In more general terms, within cardiovascular medicine, CRISPR may be applied to monogenic diseases; however, the promise of polygenicity and complexity presents very real barriers to its use in conditions like hypertension, including delivery and specificity concerns regarding off‐target effects, as well as ethical considerations [128, 129].

Phage therapy is yet an experimental approach and has not shown clinically proven efficacy in the management of hypertension. Bacteriophages may offer strain‐specific precision, with theoretical potential to modulate gut microbes in BP regulation; however, current studies have provided evidence on related conditions, such as obesity and type 2 diabetes. Essential challenges are relevant microbial targets, effective phage cocktails, safety and regulatory issues [130].

*Clostridium butyricum* –pMTL007–GLP‐1 (CB‐GLP‐1) is a genetically modified probiotic that continuously expresses the hormone GLP‐1 and therefore provides both endocrine and microbiome modulating effects. CB‐GLP‐1 significantly lowered BP in SHR rats, improved cardiac remodelling markers ACE2, AT2R and ANP as well as AMPK/mTOR signalling pathways, and restored gut dysbiosis by increasing *Lactobacillus* and reducing the abundance of *Porphyromonadaceae*. Conventional probiotics or dietary approaches to the gut microbiota do not provide sustained activity for systemic antihypertensive effects through their associated GLP‐1 action, as does CB‐GLP‐1. FMT and synbiotics are not engineered to express hormones, whereas through targeted dual mechanisms of action, CB‐GLP‐1 outperforms them, achieving long‐lasting therapeutic effects. In this way, findings like those above make CB‐GLP‐1 a next‐generation live biotherapeutic with two mechanistic actions, based on its preclinical performance in models of hypertension [131, 132, 133].

## 5 Preclinical and Clinical Evidence: Microbiome Modulation and Blood Pressure Control

Following the elucidation of the complex interplay between gut microbiota and BP regulation, microbiome‐targeted interventions emerged as a focus of investigation. The majority of microbiome‐targeted interventions investigated to date have primarily focused on the administration of prebiotics, probiotics and synbiotics, as well as FMT. These strategies aim to modulate gut microbial composition and activity, thereby influencing host metabolic and cardiovascular outcomes, including BP regulation. Numerous preclinical studies, clinical investigations and RCTs have explored the impact of gut microbiome modulation on BP regulation. A summary of these RCTs is presented in Table 1.

**TABLE 1:** Randomised controlled trials investigating the impact of gut microbiome modulation on blood pressure regulation.

Author, year	Population	Design	Time‐horizon	Outcomes
Irwin et al., 2018 [134]	Healthy individuals *n* = 38 BMI = 23.6 kg/m^2^	1:1:1:1 randomization (Prebiotics including Larch gum from *Larix occidentalis* ; Probiotics including *Lactobacillus acidophilus* (NCFM) and *Bifidobacterium lactis* ; Bi‐07; Prebiotics + probiotics; placebo)	8 weeks	No significant changes in either SBP or DBP were observed across any of the study arms
Verhaar et al., 2024 [135]	Hypertension cases *n* = 23 BMI = 24.6 kg/m^2^	1:1 randomization (Oral butyrate vs. placebo)	4 weeks	Oral butyrate administration led to a significant increase in daytime SBP (+9.63 mmHg) and DBP (+5.08 mmHg)
Jama et al., 2023 [136]	Treatment naive hypertension patients *n* = 20 BMI = *N*/A	1:1 randomization (Acetylated and butyrylated HAMSAB vs. placebo)	3 weeks of intervention and 3 weeks of wash out period	HAMSAB administration resulted in a 6.1 mmHg reduction in 24‐h, daytime and nighttime SBP compared to the placebo group
Pena et al., 2014 [137]	Obese patients *n* = 38 BMI = 36.7 kg/m^2^	1:1 randomization (Synbiotic consisting 8 g oligofructose +1 g of lyophilized *Bifidobacterium lactis* Bb12 (1010 CFU/g) vs. 9 g maltodextrin as placebo, twice a day)	6 weeks	A greater reduction in both SBP (−8 mmHg vs. −1 mmHg) and DBP (−5 mmHg vs. −0.5 mmHg) in synbiotic arm compared to placebo arm
Hadi et al. 2019 [138]	Overweight or obese patients *n* = 60 BMI ~31 kg/m^2^	1:1 randomization (synbiotics consisting *Lactobacillus acidophilus, Lactobacillus casei and Bifidobacterium bifidum* plus inulin vs. placebo)	8 weeks	No significant decline in SBP and DBP at the end of follow‐up
Rabiei et al., 2015 [139]	Patients with MetS *n* = 46 BMI ~32.4 kg/m^2^	1:1 randomization (Two probiotic capsulse vs. two placebo capsules)	3 months	Both SBP and DBP significantly reduced in both arms at the end of follow‐up, however the change in SBP and DBP were comparable among the two arms
Zolgadhrpour et al., 2024 [140]	Patients with MetS *n* = 44 BMI ~29.5 kg/m^2^	1:1 randomization (Synbiotic yogurt vs. regular yogurt)	12 weeks	Synbiotic yogurt led to a slight reduction in SBP, with more favourable changes compared to the regular yogurt group
Luangphiphat et al., 2025 [92]	Patients with MetS *n* = 58 BMI ~27.7 kg/m2	1:1 randomization (Probiotics consisting *Lacticaseibacillus paracasei* MSMC39‐1 and *Bifidobacterium animalis* TA‐1 vs. placebo)	12 weeks	Significant reduction in both SBP and DBP were observed in probitoics group, however the changes in BP were comparable across the two arms
Cicero et al., 2021 [141]	Elderly patients with MetS *n* = 60 BMI = 27.4 kg/m^2^	1:1 randomization (Synbiotic including *Lactobacillus plantarum, Lactobacillus acidophilus* and *Lactobacillus reuteri* with active prebiotics vs. placebo)	2 months	A significant reduction in mean BP with use of synbiotic
Xavier‐Santos et al., 2018 [142]	Patients with MetS *n* = 45 BMI ~32.5 kg/m^2^	1:1 randomization (Synbiotic diet mousse containing *Lactobacillus acidophilus* La‐5 vs. placebo)	8 weeks	No significant decline in SBP and DBP at the end of follow‐up
Asemi et al., 2022 [143]	Diabetic patients *n* = 62 BMI ~30 kg/m^2^	1:1 randomization (Synbiotic including L.sporogenes and inulin vs. control food)	6 weeks	— Both SBP and DBP reduced in both arms — Both SBP and DBP changes were comparable among the groups
Farrokhian et al., 2019 [144]	Overweight diabetic patients with CAD *n* = 60 BMI ~31 kg/m^2^	1:1 randomization (Synbiotic comprisng *Lactobacillus acidophilus* strain T16, *Lactobacillus casei* strain T2, *Bifidobacterium bifidum* and inulin vs. placebo)	12 weeks	A modest, non‐significant reduction in both SBP and DBP was observed in the synbiotic group, with changes remaining comparable across all groups
Attaye et al., 2025 [145]	Patients with prediabetes *n* = 98 BMI ~32.5 kg/m^2^	1:1 randomization (Encapsulated cells of *A. soehngenii* CH‐106 vs. placebo)	3 months	12 weeks of *A. soehngenii* supplementation resulted in non‐significant, slight decrease in both SBP and DBP and change in DBP was significantly greater in *A. soehngenii* group compared to group received placebo
Ekhlasi et al., 2017 [146]	Patients with NAFLD *n* = 60 BMI ~28	1:1:1:1 randomization (Synbiotic, alpha tocopherol, symbiotic + alpha tocopherol; placebo)	8 weeks	Compared to placebo, combined synbiotic and kg/m^2^alpha‐tocopherol treatment, as well as each administered individually, led to significant reductions in SBP (−17.07 ± 2.1 mmHg, −16.07 ± 3.56 mmHg, −1.73 ± 2.25 mmHg and − 1.55 ± 3.01 mmHg, *p* = 0.01)
Bakhshimoghaddam et al., 2018 [147]	Patients with NAFLD *n* = 102 BMI = 31.2 kg/m^2^	1:1:1 randomization (300 g synbiotic yogurt containing 108 CFU Bifidobacterium animalis/mL and 1.5 g inulin; conventional yoğurt; control group)	24 weeks	Significant reduction in SBP in cases consumed synbiotic yogurt compared to control group No significant differences in terms of DBP change across the arms
Esmaeilinezhas et al., 2020 [148]	Patients with PCOS *n* = 92 BMI ~26.25 kg/m^2^	1:1:1:1 randomization (Pomegranate juice; synbiotic beverage; synbiotic pomegranate juice; placebo beverage)	8 weeks	A significant reduction in both SBP and DBP in synbiotic pomegranate juice and pomegranate juice arms were observed at the end of follow‐up and these reductions were significantly greated compared to control group
Karimi et al., 2020 [149]	Patients with PCOS *n* = 99 BMI = 32.4 kg/m^2^	1:1 randomization (Synbiotic consisting *Lactobacillus acidophilus, Lactobacillus casei, Lactobacillus bulgaricus, Lactobacillus rhamnosus, Bifidobacterium longum, Bifidobacterium breve, Streptococcus thermophilus* and prebiotic Inulin vs. placebo)	12 weeks	A significant reduction in DBP was observed in the synbiotic group, which exceeded that of the placebo arm. In contrast, changes in SBP were comparable between groups
Talebi et al., 2020 [150]	Patients with hypothyroidism *n* = 60 BMI ~27 kg/m^2^	1:1 randomization (Synbiotic including *Lactobacillus Casei, Lactobacillus Acidophilus, Lactobacillus Rhamnosus, lactobacillus Bulgaricus, Bifidobacterium Breve, Bifidobacterium Longum, Streptococcus Thermophilus with* fructooligosaccharide as a prebiotic vs. placebo)	8 weeks	No significant alterations in either SBP or DBP were detected within any group or between groups
Fan et al., 2025 [119]	Hypertensive patients *n* = 124 BMI ~26.5 kg/m^2^	1:1 randomization (Faecal microbiota transplantation vs. placebo)	1 month (Primary endpoint)	A 6.2 mmHg decrease in SBP after 30 days, whereas the change in SBP is comparable with the placebo arm
Mocanu et al., 2021 [151]	Patients with severe obesity *n* = 70 BMI ~26.5 kg/m^2^	1:1:1:1 randomization (FMT‐HF; FMT‐LF; HF; LF)	12 weeks	At week 12, SBP decreased in FMT‐HF and LF groups and DBP decreased in FMT‐HF, FMT‐LF and LF groups
Leong et al., 2020 [152]	Obese adolescents *n* = 87 BMI ~37.8 kg/m^2^	1:1 randomization (Single course of oral encapsulated faecal microbiome from 4 healthy lean donors vs. placebo)	6 weeks	No significant changes were observed in office SBP and DBP levels. ABPM SBP decreased in placebo arm. Failed to show benefit of FMD on BP reduction
Hartstra et al., 2020 [153]	Adults with MetS *n* = 24 BMI ~37.7 kg/m^2^	1:1 randomization (FMT vs. butyrate)	4 weeks	Plethysmographic blood pressure measurements showed no significant changes between or within groups pre‐ and post‐intervention

Abbreviations: ABPM, ambulatory blood pressure measurement; BMI, body mass index; CAD, coronary artery disease; DBP, diastolic blood pressure; FMT, faecal microbiome transplantations; HAMSAB, high‐amylose maize starch; HF, high fermentable fibre; LF, low fermentable; MetS, metabolic syndrome; NAFLD, non‐alcoholic fatty liver disease; PCOS, polycystic ovary syndrome; SBP, systolic blood pressure.

### 5.1 Prebiotics, Probiotics and Synbiotics

Multiple preclinical studies have examined the effects of gut microbiome modulation; however, the findings remain inconclusive and frequently inconsistent, reflecting variability in study designs, intervention strategies and biological responses. In deoxycorticosterone acetate‐salt‐induced hypertensive animal models, the ingestion of a high‐fibre diet, as well as the exogenous administration of acetate—a SCFA endogenously synthesised by gut microbiota through fibre fermentation—conspicuously attenuated BP and conferred substantive protection to target organ [35]. Another study demonstrated that the administration of a probiotic formulation containing *Bifidobacterium breve* and *Lactobacillus fermentum* effectively prevented the elevation of BP and the onset of endothelial dysfunction in SHR rats [63]. Other animal studies have yielded conflicting findings regarding the impact of butyrate—a SCFA—on BP, with some demonstrating a BP lowering effect, while others reported incongruent or null outcomes [135].

Several RCTs have explored the effects of microbiome modulation by prebiotics, probiotics, or synbiotics on BP across diverse populations. A small‐scale RCT in healthy volunteers failed to demonstrate any BP‐lowering effects of prebiotics (Larch Gum from *Larix occidentalis* ) or probiotics containing *Lactobacillus acidophilus* and *Bifidobacterium lactis* after 8 weeks of follow‐up [134]. Another RCT demonstrated that a 3‐week administration of acetylated and butyrylated high‐amylose maize starch significantly reduced 24‐h, daytime, and nighttime SBP by 6.1 mmHg compared to placebo in individuals with hypertension [136]. Conversely, a trial evaluating the effects of oral butyrate in patients with hypertension reported a significant increase in both SBP and DBP after 4 weeks of follow‐up [135].

The antihypertensive potential of synbiotics was evidenced in individuals with obesity. A 6‐week intervention comprising 8 g oligofructose and 1 g lyophilized *Bifidobacterium lactis* Bb12 (10^10^ CFU/g) elicited a more pronounced reduction in both SBP and DBP relative to placebo [137]. In contrast, another RCT in overweight and obese individuals found no significant benefit of an 8‐week synbiotic regimen—comprising *Lactobacillus acidophilus* , *Lactobacillus casei* , *Bifidobacterium bifidum* and inulin—on either SBP or DBP reduction [138].

A RCT carried out in individuals fulfilling metabolic syndrome criteria demonstrated significant reductions in both SBP and DBP across both groups; however, the extent of reduction did not differ between the intervention and control arms [139]. In a study involving 44 individuals with metabolic syndrome, 12 weeks of synbiotic yogurt supplementation did not lead to a statistically significant reduction in SBP; however, the change in SBP was more favorable compared to the control group receiving regular yogurt [140]. Luangphiphat et al. delineated that synbiotic supplementation including *Lacticaseibacillus paracasei MSMC39‐1* and *Bifidobacterium animalis* resulted in a significant reduction in SBP and DBP in cases with metabolic syndrome; however, the BP changes were similar compared to the placebo arm [92]. Synbiotic supplementation also was shown to reduce mean arterial pressure among elderly patients with metabolic syndrome [141]. Conversely, another study involving patients with metabolic syndrome did not demonstrate any BP‐lowering effect from synbiotic supplementation with *Lactobacillus acidophilus* La‐5 and inulin [142].

RCTs conducted in patients with diabetes have not demonstrated a significant benefit of microbiome modulation on BP regulation. In one such study, a 6‐week intervention with a synbiotic containing *Lactobacillus sporogenes* (1 × 10^7^ CFU) and 0.04 g of inulin as a prebiotic resulted in significant reductions in both SBP and DBP among individuals with diabetes. However, the magnitude of BP reduction was comparable between the synbiotic and control groups [143]. Farrokhian et al. reported a modest reduction in both SBP and DBP among overweight individuals with diabetes; however, the changes in BP were not significantly different between the group receiving a synbiotic formulation containing *Lactobacillus acidophilus* , *Lactobacillus casei* , *Bifidobacterium bifidum* and inulin, and the placebo group [144]. Moreover, a study evaluated the effect of 12 weeks of *Anaerobutyricum soehngenii* supplementation on BP in cases with prediabetes and found that supplementation was associated with a greater reduction in DBP compared to placebo [145].

The effect of gut microbiome modulation on BP changes was assessed also in patients with NAFLD and polycystic ovary syndrome (PCOS). A trial demonstrated that an 8‐week synbiotic intervention—administered with or without alpha‐tocopherol—resulted in significantly greater reductions in SBP compared to placebo in patients with NAFLD. In contrast, DBP remained unchanged, with no significant differences observed between groups [146]. Another study randomised 102 patients with NAFLD into three arms: a synbiotic yogurt group containing 10^8^ CFU/mL *Bifidobacterium animalis* and 1.5 g inulin, a conventional yogurt group, and a control group. After 24 weeks of intervention, SBP reduction was significantly greater in the synbiotic yogurt group compared to controls, while DBP changes were comparable across all groups [147].

An 8‐week intervention with either synbiotic pomegranate juice or plain pomegranate juice led to significant reductions in both SBP and DBP in patients with PCOS, with both interventions yielding significantly greater improvements compared to the control group [148]. Another study revealed that the synbiotic intervention elicited a statistically significant reduction in DBP, with the magnitude of decline surpassing that observed in the placebo group in patients with PCOS. However, the change in SBP did not differ significantly between the intervention and placebo arms, indicating a selective effect on diastolic parameters [149]. The effect of synbiotic supplementation was assessed in a trial involving 60 patients with hypothyroidism. However, the intervention did not result in any significant reductions in either SBP or DBP [150].

Owing to the discordant findings reported across individual studies, multiple meta‐analyses have been undertaken to rigorously ascertain the effect of gut microbiome modulation by synbiotics, probiotics and prebiotics on BP regulation. A meta‐analysis including 11 RCTs concluded that synbiotic interventions elicited a marked reduction in SBP without concomitantly altering DBP. Subgroup analyses delineated that the SBP lowering impact of synbiotics was accentuated in studies of extended duration (≥ 12 weeks), among younger cohorts (< 50 years) and when administered in supplemental form [107]. Another meta‐analysis encompassing seven studies with a total of 653 participants demonstrated a beneficial effect of *Lactobacillus plantarum* ‐containing probiotic formulations on BP reduction. Specifically, these probiotics were associated with a modest decrease in SBP (weighted mean difference [WMD]: −1.58 mmHg; 95% CI: −3.05 to 0.11) and a statistically significant reduction in DBP (WMD: −0.92 mmHg; 95% CI: −1.49 to −0.35) [154]. An umbrella meta‐analysis encompassing 15,494 participants demonstrated significant reductions in SBP (−1.96 mmHg) and DBP (−1.28 mmHg) following probiotic supplementation, with more pronounced SBP reductions observed in individuals aged > 50 years, those with type 2 diabetes or hypertension, and interventions lasting ≤ 10 weeks [155].

### 5.2 Faecal Microbiota Transplantation

FMT has been established as a therapeutic modality in various gastrointestinal disorders, including *Clostridium difficile* infection and inflammatory bowel disease (IBD) [156]. Its potential application in the management of hypertension has been explored in both preclinical and clinical settings. Experimental studies demonstrated that transplantation of microbiota from hypertensive humans or hypertensive rats into normotensive recipient rats induced a notable elevation in BP [25, 157]. In a clinical observational study, washed microbiota transplantation from normotensive, healthy donors to hypertensive individuals resulted in a significant reduction in both SBP (−5 mmHg) and DBP (−7 mmHg) at the time of discharge [158]. The combined effect of fibre supplementation and FMT was investigated in individuals with severe obesity. SBP was significantly reduced in participants receiving low‐fermentable (LF) fibre alone and those receiving FMT in combination with high‐fermentable (HF) fibre. DBP showed significant reductions in the FMT‐HF fibre, FMT‐LF fibre and LF fibre groups [151]. Conversely, a RCT evaluating FMT from normotensive donors demonstrated a 6.2 mmHg decrease in SBP after 30 days; however, this reduction was not statistically distinguishable from that observed in the placebo arm, indicating a comparable between‐group effect [119]. Moreover, another RCT carried out in obese adolescents failed to demonstrate a BP–lowering effect following a single course of FMT, indicating potential age‐ or population‐specific variability in therapeutic response [152]. FMT did not lead to a significant reduction in BP and, when compared to butyrate administration, did not demonstrate any additional antihypertensive benefit after a 4‐week follow‐up in adults with metabolic syndrome [153].

The majority of current evidence supports the beneficial role of gut microbiome modulation in BP regulation. However, some studies have reported inconsistent or inconclusive findings, which may be attributed to variations in the specific microbial strains used, differences in probiotic formulations, heterogeneity in study populations, or methodological limitations such as small sample sizes and short intervention durations. Consequently, there is a need for well‐designed, large‐scale RCTs to clarify the precise effects of gut microbiota modulation on BP outcomes and to establish standardised protocols for clinical application.

## 6 Methodological Challenges and Research Gaps

Obesity and hypertension, two interlinked global health challenges, require urgent attention. Despite the range of therapeutic strategies available, from lifestyle interventions to pharmacotherapy and bariatric surgery, the lack of clarity on the shared pathophysiology and treatment of these conditions is a pressing issue that needs to be addressed. A major methodological challenge is the heterogeneity of study populations and intervention protocols. Clinical trials often vary in their inclusion criteria, baseline characteristics and outcome definitions, which limits their comparability and generalizability. For example, while GLP‐1 receptor agonists and dual agonists, such as tirzepatide, show concurrent benefits for weight and BP reduction, the underlying mechanisms—beyond weight loss—are not yet fully understood. These may include effects on endothelial function, natriuresis, or neurohormonal modulation, but robust mechanistic studies are lacking [159, 160, 161].

The gut microbiome has emerged as a potential upstream determinant of both obesity and hypertension. However, most studies focus narrowly on bacterial taxa, neglecting the broader microbial ecosystem. Recent research highlights the significance of the gut mucosa‐associated microbiota, which may provide more stable and disease‐specific signatures than faecal samples. A study on treatment‐naïve patients with IBD and irritable bowel syndrome revealed that the mucosal microbiota is spatially homogeneous and more effective than faecal microbiota in distinguishing disease phenotypes. This finding suggests that mucosal sampling, though invasive, may be critical for understanding host‐microbiota interactions relevant to metabolic and cardiovascular diseases [162].

Moreover, the role of non‐bacterial microbiota—fungi (mycobiome), viruses (virome), archaea (archaeome) and eukaryotic parasites—remains underexplored. Findings on the non‐bacterial microbiome in IBD highlight that these communities are dynamic, interact with bacterial populations, and influence host immunity and metabolism. For instance, fungal dysbiosis, characterised by an increase in *Candida* and a decrease in *Saccharomyces cerevisiae* , has been linked to inflammatory responses. Similarly, alterations in the gut virome, particularly an expansion of *Caudovirales bacteriophages*, have been associated with IBD and may reflect broader dysbiotic states relevant to obesity and hypertension. Methanogenic archaea, such as *Methanobrevibacter smithii* , which modulate fermentation efficiency and energy harvest, are also implicated in metabolic disorders and may influence gut transit and inflammation [163].

Despite these insights, methodological limitations persist. Microbiome studies often suffer from inconsistent sampling protocols, variable sequencing platforms and inadequate bioinformatics pipelines. The lack of standardised methods hampers reproducibility and cross‐study comparisons. Additionally, most microbiome analyses are cross‐sectional, limiting causal inference. Longitudinal studies in treatment‐naïve populations are necessary to track changes in microbiota over time and in response to interventions [161].

Another gap lies in biomarker discovery. While multiplex proteomic platforms enable high‐throughput screening of inflammatory and metabolic proteins, they often lack absolute quantification and exclude low‐abundance markers. Moreover, many identified biomarkers, such as IL‐17A and TNFRSF9, are not disease‐specific and may reflect general inflammatory states rather than disease‐specific conditions. Integrating multi‐omics data—combining proteomics, metabolomics and microbiomics—could enhance specificity, providing a more comprehensive understanding of the complex interplay between host physiology and the gut microbiome. However, this approach remains technically and analytically challenging. Psychosocial factors, including chronic stress, are also underrepresented in current research. Stress influences neurohormonal pathways and gut‐brain axis function, yet few studies incorporate stress biomarkers or assess the impact of stress‐reduction interventions. This is a critical oversight given the known links between stress, microbiota composition and cardiometabolic risk. Interventions such as mindfulness‐based stress reduction programs or cognitive‐behavioural therapy could be incorporated into research to address this gap [161, 164, 165, 166].

Finally, outcome measures across studies are inconsistent. Definitions of treatment success, such as hypertension remission post‐bariatric surgery, vary widely. Moreover, weight loss is often used as a proxy for health improvement, despite evidence that metabolic and vascular benefits may not correlate linearly with weight reduction [161].

In conclusion, advancing research on obesity and hypertension requires adopting a more holistic and standardised approach. This approach should involve the harmonisation of study designs and outcome definitions to ensure consistency and comparability across research efforts. It is essential to conduct longitudinal and mechanistic studies in diverse populations, particularly those who have not yet undergone treatment. Integrating mucosal and faecal microbiota profiling will provide a deeper understanding of the microbial influences. Additionally, research should include the study of non‐bacterial microbiota as well as incorporate multi‐omics data to capture the complexity of biological interactions. Finally, it is important to consider psychosocial and environmental factors, recognising their significant role in the development and progression of these conditions.

Only through such comprehensive strategies can the complex interplay between host physiology and the gut microbiome be better understood. Translational biomedical researchers, clinicians and academics in metabolic diseases, cardiology and applied microbiology have a crucial role in developing and implementing these strategies to address the research gaps in obesity and hypertension.

## 7 Future Directions: Precision Microbiome‐Based Interventions for Obesity‐Induced Hypertension

Obesity‐induced hypertension represents a significant global health challenge, with its pathophysiology intricately linked to metabolic dysfunction, chronic low‐grade inflammation, endothelial impairment and neurohormonal alterations. In recent years, the gut microbiome has emerged as a critical regulator of BP homeostasis. A growing body of evidence implicates gut dysbiosis in the initiation and progression of both obesity and hypertension. These insights have spurred growing interest in microbiome‐targeted therapeutic strategies as a novel and potentially modifiable intervention point.

The application of precision medicine, underpinned by multi‐omic technologies, is poised to advance this field by facilitating the development of individualised microbiome‐based interventions. The gut microbiome of patients may be significantly influenced by coexisting comorbidities and concomitant medication use, both of which can alter microbial composition and function. These factors contribute to inter‐individual variability in response to microbiome‐targeted interventions, indicating that a ‘one‐size‐fits‐all’ approach is unlikely to be effective for managing hypertension through uniform microbiome modulation strategies. By integrating host genomic, transcriptomic, proteomic, metabolomic and microbiomic data, it becomes possible to characterise the complex host–microbe interactions underlying obesity‐related hypertension and to tailor therapies accordingly.

Despite encouraging findings from preclinical models, RCTs and meta‐analyses, the clinical efficacy of gut microbiome modulation remains variable and often inconclusive. This inconsistency likely reflects heterogeneity in study designs, microbial strains or formulations used, duration and dosage of interventions, and differences in outcome measures. Importantly, inter‐individual variability—including baseline gut microbiota composition, host genetic predisposition, immune status, dietary patterns and lifestyle factors—also plays a substantial role in shaping therapeutic responses. These complexities underscore the limitations of conventional, uniform treatment strategies and highlight the necessity of adopting precision‐based approaches.

Future research should focus on stratifying patient populations based on microbial and host‐related biomarkers to optimise intervention efficacy. Embracing precision medicine frameworks will be essential to developing reliable, reproducible and clinically effective microbiome‐targeted therapies for obesity‐induced hypertension. Rigorously designed RCTs with adequately powered sample sizes are essential to elucidate the specific effects of various modalities of gut microbiome modulation on BP regulation. Moreover, both the composition of the gut microbiome and the physiological responses to microbiome‐targeted interventions may vary across different ethnic groups and geographical regions, underscoring the necessity for multinational studies to ensure the generalizability and applicability of findings across diverse populations.

Modulation of the gut microbiome may exert effects beyond the regulation of BP by also influencing hypertension‐associated target organ damage, including vascular remodelling, cardiac hypertrophy and renal impairment. These potential protective mechanisms, mediated through microbiota‐derived metabolites, immune modulation and inflammatory pathways, warrant further investigation. Future studies should emphasise the evaluation of microbiome‐targeted interventions not only on hemodynamic parameters but also on the prevention or attenuation of end‐organ damage to comprehensively assess their therapeutic potential in hypertension management.

## 8 Conclusion: Charting a Gut‐Centric Path Forward in Obesity‐Induced Hypertension

Recent findings have brought to light the significant role of the gut microbiome in the development of obesity‐induced hypertension, presenting it as a promising avenue for therapeutic intervention. Obesity is now understood not only as a metabolic disorder but also as a condition of chronic low‐grade inflammation, with gut dysbiosis playing a central role. Changes in microbial composition can compromise gut barrier function, trigger endotoxemia and influence host metabolism and immune responses—factors closely associated with high BP.

Research has demonstrated the potential of specific probiotic strains, particularly those in multispecies formulations, in restoring a healthy balance to the gut microbiome, reducing systemic inflammation and enhancing metabolic parameters. For example, the addition of *Lactobacillus* and *Bifidobacterium* strains has shown promising results in improving glucose regulation and reducing inflammatory markers in older adults, suggesting potential cardiovascular benefits. Furthermore, dietary choices such as the Mediterranean diet, which is rich in fermentable fibres and polyphenols, can promote a diverse and robust microbiota, thereby strengthening the gut‐heart connection.

While the potential of the gut microbiome as a therapeutic target is indeed promising, it is crucial to maintain a balanced perspective. The intricate nature of host‐microbiome interactions, the variability between individuals and the influence of factors such as diet, age and comorbidities all call for a cautious approach. Current clinical trials, often lacking in scale, duration and diversity, and with a dearth of standardised protocols and mechanistic insights, underscore the need for a balanced and realistic view of the field.

To truly harness the gut microbiome in combating obesity‐induced hypertension, robust, longitudinal and inclusive research is imperative. Integrating multi‐omics approaches, personalised nutrition and microbiota‐targeted interventions could pave the way for precision therapeutics. As we chart this gut‐centric path forward, it's crucial to remember that collaboration across disciplines and populations will be key to translating microbiome science into meaningful clinical outcomes.

## Author Contributions

Conceptualization: Andrej Belančić; investigation: All authors; project administration: Andrej Belančić; visualisation: Almir Fajkić and Lejla Alić; writing – original draft: All authors; writing – review and editing: All authors; supervision: Bojan Jelaković.

## Funding

The authors have nothing to report.

## Conflicts of Interest

The authors declare no conflicts of interest.

## Data Availability

The authors have nothing to report.
